# An Easy Procedure to Quantify Anticoagulant Rodenticides and Pharmaceutical Active Compounds in Soils

**DOI:** 10.3390/toxics9040083

**Published:** 2021-04-10

**Authors:** Andrea Acosta-Dacal, Cristian Rial-Berriel, Ricardo Díaz-Díaz, María del Mar Bernal-Suárez, Manuel Zumbado, Luis Alberto Henríquez-Hernández, Octavio P. Luzardo

**Affiliations:** 1 Toxicology Unit, Research Institute of Biomedical and Health Sciences (IUIBS), Universidad de Las Palmas de Gran Canaria, Paseo Blas Cabrera s/n, 35016 Las Palmas de Gran Canaria, Spain; andrea.acosta@ulpgc.es (A.A.-D.); cristian.rial@ulpgc.es (C.R.-B.); manuel.zumbado@ulpgc.es (M.Z.); luis.henriquez@ulpgc.es (L.A.H.-H.); 2 Department of Environmental Analysis, Technological Institute of the Canary Islands, C/Los Cactus No 68, Polígono Industrial de Arinaga, Agüimes, 35118 Las Palmas, Spain; rdiaz@itccanarias.com (R.D.-D.); mbernal@itccanarias.org (M.d.M.B.-S.); 3 Spanish Biomedical Research Center in Physiopathology of Obesity and Nutrition (CIBERObn), Instituto de Salud Carlos III, 28029 Madrid, Spain

**Keywords:** brodifacoum, flocoumafen, veterinary drugs, QuEChERS, LC-MS/MS, forensic analysis

## Abstract

A modified QuEChERS (quick, easy, cheap, effective, rugged, and safe) extraction was validated for the extraction of seven coumarin anticoagulant rodenticides (ARs) and 36 pharmaceutical active compounds (PhACs) residues in soil samples using liquid chromatography tandem mass spectrometry (LC-MS/MS). The aim of this work was to develop a method for the monitoring of these compounds in agricultural lands as well as in forensic applications for the determination of ARs poisoning. As far as is known, this is the first time that a QuEChERS-based method is used for the extraction of ARs in soil, as well as on such a quantity of PhACs. A matrix effect study was carried out on samples of soil devoted to agriculture in the midland area of the Canary Islands (clay loam type). It was in house validated (accuracy, precision, and linearity) at seven spiked levels between 0.5 and 50 ng g^−1^. The limits of quantification (LOQ) ranged between 0.5 and 50.0 ng g^−1^ and the limits of detection (LOD) ranged from 0.024 to 6.25 ng g^−1^. The method was then successfully used for both the determination of the target analytes in the soils from the agricultural plots that had been irrigated with regenerated water, and in the soil collected from underneath wild bird carcasses (which had been the subject of forensic investigation).

## 1. Introduction

Soil is a very vulnerable pollution receptor environment from where pollutants can be emitted to other environmental compartments such as the atmosphere, ground or surface water, and biota [1]. Contaminated soil presents a large number of problems that may include the destruction of ecosystems, agricultural productivity losses, the contamination of groundwater, and danger to human and animal life due to the accidental ingestion of soil or consumption of food that has grown on contaminated soil. Agricultural soil contamination can be direct and intended, thorough the application of pesticides to crops, or indirect and nonintended, mainly due to irrigation using regenerated waters and the use of compost of wastewater treatment plants (WWTPs) sludge. Both water and sewage sludge may contain various contaminants of emerging concern (CECs), including anticoagulant rodenticides (ARs) and pharmaceutical active compounds (PhACs).

ARs are the most widely used rodent control agent in agriculture (usually in postharvest plants and warehouses), livestock farming, and in domestic and urban applications [2]. These substances act by preventing the formation of clotting factors by blocking the vitamin K cycle and, thus, causing spontaneous internal bleeding [3]. The first generation of ARs (FGARs) started with warfarin, soon followed by coumatetralyl, chlorophacinone, and diphacinone [4]. Following the widespread development of rodent resistance to FGARs, a second and more toxic generation of compounds (SGARs), including flocoumafen, difethialone, difenacoum, bromadiolone and brodifacoum, were developed in the 1970s [5]. On the other hand, some anticoagulants, such as warfarin or its analogues, are used in the medical treatment of blood hypercoagulability disorders [6]. Taking this into account, ARs can reach wastewater through runoff after agricultural and livestock farming applications, bait solubilization in urban infrastructures or through urinary excretion from medical treatments [7,8].

PhACs are essential for the prevention and treatment of disease in humans and animals [9,10]. After administration, these active substances are absorbed and can be either metabolized or converted back into active ingredients [11]. A part of them is excreted through urine or feces, and may enter WWTPs and end up in environmental compartments as both metabolites and parent compounds [12,13]. The presence of PhACs in the environment can favor the appearance and the development of various adverse effects, such as antibiotic-resistant microorganisms, even at low levels due to continuous exposure to antimicrobials [13].

Once in WWTPs, both ARs and PhACs can be degraded, accumulate in sewage sludge or remain in the water, even after tertiary treatments, which are released to effluent receiving waters or used to irrigate crops [14]. In addition, due to their hydrophobicity, ARs are more likely to accumulate in sewage sludge, which is commonly used to make compost [15] while PhACs can be found in both media depending on their physicochemical properties [16]. Similarly, ARs are relatively stable and have a moderate to high octanol/water partition coefficient (K_OW_) allowing them to associate with natural soil organic matter [17,18]. As for the fate of PhACs, depending on their interaction with soil matrix components, they may remain in or be transported to groundwater and surface water through surface runoff, infiltration/percolation [12,19]. As seen, the amounts of ARs and PhACs that may eventually accumulate in soils through irrigation with regenerated wastewater and/or through sludge and manure spreading or from direct bait application on fields, should be a matter of concern.

Both PhACs and ARs may pose a high risk to wildlife due to their potential for bioaccumulation and transfer through trophic webs. Although ARs are intended to control rodents, baits may be consumed by other nontarget species such as granivorous birds (primary exposure). Similarly, predators or scavengers may consume rodents and nontarget animals that have already been exposed to ARs (secondary exposure) [20]. Moreover, ARs baits are often intentionally used to poison and kill not rodents but other animals. Likewise, the exposure of wild animals to PhACs can occur in several ways: contaminated water [21], agricultural soils, plants and arthropods [22,23]. The carcasses of farm animals that have been previously medicated, as well as their slurry, can also be a relevant source of exposure to pollutants for wildlife [16,24]. In such cases, exposure is usually monitored by blood or liver tissue samples from dead animals [25,26]. However, it is quite usual to find only the remains of animal carcasses and not being able to collect tissue or fluid samples. Therefore, for these specific cases, an extraction method to test the soil sampled under the carcass may be an appropriate way to prove exposure to these compounds due to the capacity of the soil organic matter to retain them after being leached from the carcasses.

There are very few studies on extraction methods for anticoagulants in soils [27,28,29,30,31,32] and, to our knowledge, none of them use the QuEChERS methodology. Although there are more methods of extracting PhACs from soils, only a small number of them use QuEChERS procedures [33,34,35,36]. The QuEChERS method consists of an acetonitrile extraction/partitioning step followed by a solid-phase dispersive extraction as a clean-up step and was first developed for the extraction of pesticides from fruits and vegetables [37]. This user-friendly methodology provides high extraction yields using reduced amounts of samples and organic solvents, which has led to its use in different matrices and analytes [38]. Modified versions of QuEChERS have been successfully applied in soils for the extraction of organic contaminants, including pesticides [39,40,41], persistent organic pollutants [42,43], as well as in the extraction of ARs and PhACs from other samples such as food [44,45], blood [46,47], sediments and sludge [48], and the liver [49,50].

Along with a robust extraction method, adequate detection analysis is necessary. ARs and PhACs have been determined mainly by liquid chromatography (LC) with different detectors such as UV [32,51], diode array (DAD) [30,52], DAD with fluorescence detectors (FLD) [53,54] and single quadruple mass spectrometry (MS) detectors [28,55]. However, the preferred option when looking for sensitivity and selectivity at trace level is liquid chromatography-tandem mass spectrometry (LC-MS/MS) [7,35,56,57], which is the best option for low-level residues that can be expected to be found in soils.

The objective of this research was to evaluate and validate a QuEChERS-based and LC-MS/MS method for the quantification of ARs and PhACs in soils, as well as their verification in samples of agricultural origin and from wildlife carcasses. The work is proposed as a scope extension of the previously optimized method for the quantification of pesticide residues in soils [41]. The proposed method can be applied in agricultural, environmental and forensic monitoring.

## 2. Materials and Methods

### 2.1. Reagents and Chemicals

Individual certified standards of ARs and PhACs (purity 95.19% to 99.9%) were acquired from Dr. Ehrenstorfer (Augsburg, Germany), Sigma-Aldrich (Augsburg, Germany) and European Pharmacopoeia Reference Standards (Strasbourg, France). Atrazine-d5, Carbendazim-d3, Coumachlor, Cyromazine-d4, Linuron-d3 and Pirimicarb-d6 (Dr. Ehrenstorfer and Sigma-Aldrich, 99.3–99.9% purity) were used as procedural internal standards (P-IS) and were maintained as described in the reference method [41]. These internal standards were added to the samples at the beginning of the procedure to account for various sources of errors throughout all stages in the method [58]. The list of the selected analytes, their classification, and their legal status are in Table 1.

LC-MS grade methanol (MeOH), acetonitrile (ACN) and formic acid (FA, HCOOH) were obtained from Honeywell (Morristown, NJ, USA). Ammonium acetate (NH_4_CH_3_CO_2_) was purchased from Fisher Scientific (Loughborough, UK). AOAC method QuEChERS salts [59] (6 g of MgSO_4_ and 1.5 g of CH_3_COONa) were acquired in commercial premixes from Agilent Technologies (Palo Alto, CA, USA). The ultrapure water was produced in the laboratory using a Gradient A10 Milli-Q System (Millipore, Bedfore, MA, USA).

### 2.2. Standard Stock Solutions and Mixes

Individual standard stock solutions of all analytes and P-IS were prepared in ACN at a concentration of 1000 μg mL^−1^. Individual intermediate solutions of 1 μg mL^−1^ were prepared for spectrometry optimization. In addition, two mixed stock solutions were prepared, one containing target ARs and the other containing the selected PhACs, at 10 μg mL^−1^ followed by a mixed working solution diluted ten times more than 1 μg mL^−1^ of each. Finally, a P-IS solution was prepared at 1 μg mL^−1^.

Both solvent and matrix-matched calibration curves were prepared with the standard working mix solution in either soil extracted with the evaluated procedure or ACN 2.5% FA diluted with ultrapure water (1:1, *v*/*v*).

All standards, working mix solutions and matrix-matched calibrators were stored in glass amber vials at −20 °C and checked periodically for stability.

### 2.3. Sample Selection and Pretreatment

The extraction method was validated on a representative type of soil, which due to its physicochemical properties, can be classified as clay loam soil. The soil was chosen from two farms dedicated to organic production and sampled at various times throughout 2020. A composite sample of at least four subsamples collected at depths between 20 and 30 cm was prepared in each sampling plot. It was then thoroughly mixed and homogenized, air-dried at room temperature and sieved (2 mm mesh) before being considered suitable for analysis.

The physicochemical properties of this composite are as follows: pH 4.88, electrical conductivity 209 μS cm^−1^, oxidizable organic carbon 2.19% (approximately 3.9% organic matter), 6% moisture and particle size distribution: 29.5% clay, 28.3% fine silt, 11.3% thick slit, 11.5% coarse sand, and 19.4% fine sand. All these parameters were determined in the facilities of the Department of Department of Animal Biology, Edaphology and Geology of the University of La Laguna (Tenerife, Spain) [41].

### 2.4. Sample Preparation

The method used for the extraction of the target analytes was previously developed and validated in our laboratory for the extraction of pesticides in clay loam soil [41,60]. In summary, the samples were prepared by weighing 10 ± 0.05 g of dried and sieved soil into a 50 mL centrifuge. All samples were spiked with the appropriate volume of the P-IS solution to achieve a concentration of 5 ng g^−1^, and recoveries and Quality Controls (QCs) were added standard mix solutions and left to stand for 1 h prior to extraction. Then, 10 mL of ACN-2.5% FA were added and vigorously shaken for 1 min. In the same way, 6 g of MgSO_4_ and 1.5 g of CH_3_COONa were added, energetically shaken for another minute and sonicated for 15 min in an ultrasonic bath operating at 50/60 Hz and 120 W (VWR, Radnor, PA, USA). After that, samples were placed 25 min in a rotatory shaker (Ovan, Barcelona, Spain). They were then centrifuged for 10 min at 4200 rpm (3175.16× *g*) in a 5804 R Eppendorf centrifuge (Eppendorf, Hamburg, Germany). An aliquot of the supernatant extract was filtered through 0.20 µm Chromafil^®^ PET filters (Macherey-Nagel, Düren, Germany) and diluted with H_2_O (1:1, *v*/*v*) before analysis in LC-MS/MS.

### 2.5. LC-MS/MS Analysis

LC-MS/MS equipment (1290 model UPLC tandem coupled to a 6460 model Triple Quadrupole mass spectrometer, Agilent Technologies, Palo Alto, CA, USA). The chromatographic column was an Agilent Poroshell 120 EC-C18 (2.1 × 100 mm, 2.7 µm), with a guard precolumn (2.1 × 5 mm, 1.8 µm) plus a and prefilter. The column oven was set at 50 °C. The mobile phases consisted of 2 mM ammonium acetate 0.1% formic acid in ultrapure water (A) and 2 mM ammonium acetate in MEOH (B). The binary gradient was set as follows: 5% B–0.5 min; 5% B–1 min; 40% B–2.5 min; 85% B–8 min; 100% B–10 to 14 min; 5% B–14.01 min. The flow rate was 0.4 mL min^−1^. The volume injected was 5 μL and the total run time was 18 min.

The parameter for the MS/MS analyzer were those described in the original method [41]. Data analysis was performed using Agilent software MassHunter Quantitative Analysis (for QQQ) version B.07.01 and MassHunter Qualitative Analysis version B.07.00.

The MS/MS conditions were optimized by injecting 5 μL of individual solutions of each compound at 1 μg mL^−1^ in ACN directly to the mass spectrometer replacing the chromatographic column with a stainless steel zero dead volume union. The mobile phases were set in isocratic mode (50:50, *v*/*v*), and these were 2 mM ammonium acetate and 0.1% FA in ultrapure water (phase A) and 2 mM ammonium acetate and 0.1% FA in methanol (B). The product ions were optimized in the MRM mode at different collision energies, from which that exhibiting the highest response was selected.

### 2.6. In House Validation Parameters

To validate the use of the proposed method and broaden it to the selected ARs and PhACs, we performed a single-laboratory validation. Since there is no a specific guidance for the analysis of ARs and PhACs residues in soil, the in house validation (hereafter validation) of the proposed method was performed following the guidelines for the analysis of pesticide residues in food and feed of the European Union [58,61].

The linear range of LC-MS/MS method was studied using calibration curves prepared in soil matrix-water (1:1, *v*/*v*), ranging from 0.024 to 50 ng g^−1^ using both correlation coefficient and Mandel test (95% confidence level) [62]. Accuracy was estimated by recovery assays (in quintuplicate) through the quantification of trueness and precision (% relative standard deviation) at 7 concentration levels: 0.5, 1, 2.5, 5, 10, 20 and 50 ng g^−1^. According to the SANTE and SANCO guides, recoveries between 70–120% are considered acceptable, when the RSDs are below 20%. Additionally, following the criteria specified in those guides the limit of quantification (LOQ) for each compound was the lowest level of the recovery experiments that met all the validation criteria: recovery between 80–120%, and RSD below 20%. On the other hand, the limits of detection (LOD) were calculated using calibration standards. For this purpose, triplicate matrix-matched calibration curves were prepared ranging from 0.024 to 100 ng g^−1^. Thus, the LOD was selected as the lowest point of the calibration curve that meets had a signal-to-noise ratio (S/N) > 3 (Peak-to-Peak algorithm) and an accuracy between 80–120%.

The identity of the compounds was based on the acquisition of two transitions in the MRM mode, one of them employed as the quantification transition (Q), and the other as the confirmation transition (q). According to the SANTE and SANCO guides an ion ratio tolerance of 30% was considered acceptable. The tolerance for the deviation of the retention time (tR) ± 0.1 with respect to that of the reference standard.

## 3. Results and Discussion

### 3.1. Optimization of LC-MS/MS Conditions

The seven target rodenticides, chloramphenicol, tolfenamic acid, and the P-IS coumachlor were determined in negative mode with precursor ions corresponding to [M − H]^−^. The remaining analytes and P-IS were analyzed in positive mode using precursor ions corresponding to [M + H]^+^ except for cefuroxime axetil, cloxacillin, eprinomectin, josamycin and penicillin V. It is known that, together with the protonated and deprotonated molecules already mentioned, the ionization process can produce a variety of adduct ions depending on the composition and concentration of mobile-phase additives and analytes, the pH, the mobile-phase flow rate, the temperature or even solvent impurities and glassware [63,64,65]. While cefuroxime axetil formed a sodium adduct [M + Na]^+^, cloxacillin, josamycin and penicillin V formed methanol adducts, which was the organic phase solvent. In the case of eprinomectin, the ion *m*/*z* 878 selected as parental for both transitions is its protonated derivative ion resulting from dehydrative aromatization [66]. In the case of brodifacoum and bromadiolone, which have Br in their chemical structures, an element with an abundant characteristic isotope distribution, transitions corresponding to ^79^Br and ^81^Br were selected. In the same way, chloramphenicol, which has two atoms of Cl in its structure, was optimized selecting the transitions corresponding to ^35^Cl and ^37^Cl. The transitions were chosen in terms of selectivity and sensitivity, selecting the most abundant ones for quantification proposals.

The parameters of the ion source were then optimized injecting standards at 100 ng mL^−1^ in ACN (in triplicate). The parameters were nebulizer and sheath gas flow and temperature, and capillary and nozzle voltages. The Agilent software (Mass Hunter Source Optimizer) was employed for this purpose.

Likewise, we also injected 20 ng mL^−1^ standards in triplicate, prepared in the same solvent, for the chromatographic condition optimization process. The final conditions selected for both the source and the chromatography were then tested with a matrix-matched standard at the same concentration. We decided to use Poroshell 120 EC-C18 column (2.1 × 100 mm, 2.7 µm; Agilent Technologies) due to the satisfactory performance for the variety of pesticide belonging to different chemical groups optimized in the previous work [41]. The mobile phase solvents and modifiers used for the compound optimization process were also set as the final phase composition to ensure the formation of the mentioned adducts. However, different concentrations and gradients of ammonium acetate (2 and 5 mM) and FA (none and 0.1%) were tested to achieve the best resolution and separation of the compounds. A higher amount of ammonium not only did not improve the sensitivity, but also increased the pressure in the column, so it was set as 2 mM. However, we eliminated FA from the organic phase as it worsens the sensitivity of ARs compounds, but we continued to use it in the aqueous phase because it was necessary for the analysis of some PhACs. This decision was made because ARs retention starts with warfarin at 7.87 min (with this combination of mobile phases and gradient) where the organic mobile phase is approximately 83% and continues to increase. This way, the acid composition in the column will decrease when the ARs are retained in the column.

The injection volume was set at 5 μL after testing from 1 to 20 μL because it produced good peak shapes while giving high responses. In addition, a higher volume would result in saturation of the MS/MS detector. The possible dilution of the extract with water was also evaluated at this point before injecting it in the LC-MS/MS equipment. The ratios tested were 1:1, 1:2, 1:5 y 1:10 (*v*/*v*) of 20 ng g^−1^ standards in ACN-2.5%FA-ultrapure water. The experiment was then repeated with standards in the soil matrix at the same dilutions. In order to achieve considerable sensitivity and selectivity, while improving the peak shape of some compounds such as chloramphenicol, dexamethasone and mebendazole, we chose to dilute the final extract 1:1, *v*/*v* with water.

**Figure 1:**
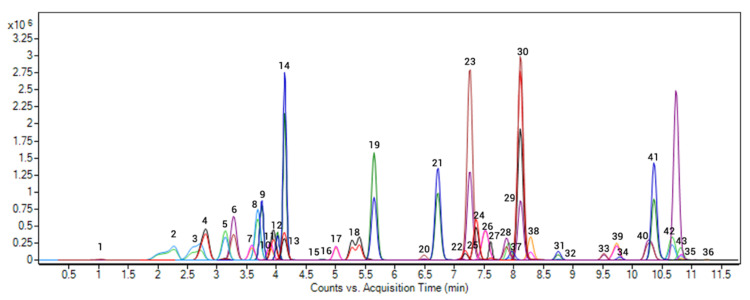
LC-MS/MS chromatograms of a blank soil sample spiked with the anticoagulant rodenticides (ARs) and the pharmaceutical active compounds (PhACs) at 50 ng g^−1^.

All the chromatography and mass spectrometry conditions are shown in Table 1. In addition, a chromatogram of a blank soil sample spiked at 50 ng g^−1^ is shown in Figure 1.

### 3.2. Matrix Effect Study

Soil is an extremely complex matrix whose components can suppress or enhance the response of the target analytes in the mass spectrometer and can therefore condition the integrity of the analysis. Bearing this in mind, a study of the matrix effect was performed. The matrix effect (ME) was evaluated comparing the slopes of calibration curves in the solvent (S_S_) and in the matrix (S_m_), which was extracted using the procedure described in Section 2.4, according to the equation:

ME (%) = (S_m_/S_S_) × 100 (1)

Hence, the effect of the matrix components on the signal is qualified as the percentage of enhancement or suppression, whether the ME values are above or below 100%, respectively. No significant matrix effects were considered when ME was between 80–120% [61].

**Figure 2:**
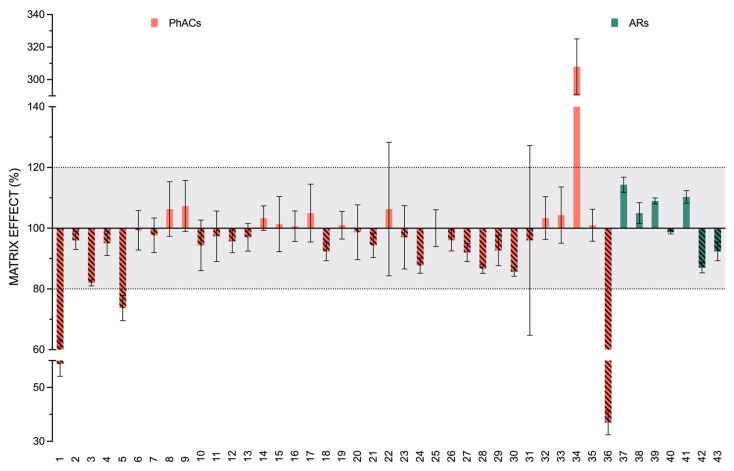
Matrix effect of the ARs and PhACs in the soil used for the validation process. Bars represent the mean recoveries and the SD of the 43 analytes. PhACs (orange) and ARs (green) are presented according to their number in Table 1. The suppression has been indicated with an oblique dashed pattern.

The calibration curves covered the range of 3.125 to 50 ng g^−1^ and were prepared either in soil matrix or ACN 2.5%FA, both diluted with ultrapure water (1:1, *v*/*v*). All curves were prepared in triplicate and adjusted to a linearity equation (y = ax + b). Figure 2 shows ME mean values and SD together with the tolerance interval in which no matrix effect is considered (in grey). As can be seen in the graph, the majority of the analytes did not present a significant matrix effect. However, moxidectin, sulfanilamide, and sulfapyridine showed strong, medium, and slightly significant signal suppression, respectively, and only tolfenamic acid showed strong signal enhancement. Although cloxacillin and imipenem are in the tolerance range of ME%, they presented a variable value with RSD% over 20%. Consequently, matrix-matched calibration was used for quantification in the subsequent experiments.

### 3.3. In House Validation Studies

The proposed extraction method was validated following the agreements stated in the “In house validation parameters” section.

Linearity response was satisfactory in the studied range for each compound, with R^2^ values higher than 0.99 and *p*-values over 0.05 after Mandel test application (Table 2). All ARs and the majority of the PhACs were satisfactorily extracted in terms of accuracy and precision from their LOQ to the highest concentration tested, with recoveries between 70–120% and RSD values below 20%, respectively. However, cloxacillin and tolfenamic acid showed recoveries above 120% for some of the concentration levels. Similarly, albendazole, dexamethasone, flunixin, oxfendazole, penicillin V, sulfacetamide, sulfametoxipiridacine, sulfamonomethoxine, sulfanilamide and sulfapyridine were recovered below 70% at some of these levels. However, all of these compounds were extracted with high reproducibility (RSD < 20%) in the range of 60–130%, which is contemplated in the SANTE guidelines for routine analysis [58]. On the other hand, other PhACs showed high variability (RSD > 20%) at 5 ng g^−1^ or below but were recovered between 70–120% which is also taken into account in these guidelines for concentration below 10 ng g^−1^ of residues in soils. The results of the recovery experiments are summarized in Table 3.

All analytes LOQs were equal to or below 20 ng g^−1^ except for imipenem with 50 ng g^−1^, which is the LOQ normally required for residues in soils according to the guidance for analytical methods for residues in soil [61]. As for the LOD, all analytes had a detection limit of 6.25 ng g^−1^ or even lower (Table 2). As mentioned above, there are very few studies that determine ARs in soil and none of them analyze difethialone residues. Moreover, the LOQ and LOD values obtained with our modified QuEChERS method are much lower than those obtained using other extraction methodologies [27,29,31] except for brodifacoum, coumatetralyl, difenacoum and flocoumafen, whose LOQs are in the same range than those obtained with a MeOH and ammonium format extraction [28]. However, it should be noted that the LOD and LOQ in those cases were established as three and ten times the S/N of the blank sample extract, respectively, while ours were calculated following the more restrictive criteria of the European Union SANTE guidance [58]. Furthermore, among the few QuEChERS-based extraction methods for PhACs in the literature for their determination in soils, our method is capable of extracting and analyzing more of these compounds. Of the compounds that coincide with those determined in the previously published methods, sulfanilamide, sulfadiazine, and sulfadimethoxine have lower LOQs in the method of Salvia et al. [35] but were determined as S/N ratio equal to 10. Similarly, Lee et al. also determined the LOQs as ten times the S/N ratio, and obtained a LOQ for sulfamethazine that was equal to ours (0.5 ng g^−1^), but the one for sulfamethoxazole was lower (0.5 vs. 2.5 ng g^−1^) [33]. Finally, Ferhi et al. determined the LOQs with the results of the validation data in a more similar way to ours for diclofenac, sulfamethoxazole, and sulfamethoxazole, our values being lower in the first two cases and slightly higher but in the same range in the latter (2.1 ng g^−1^ vs. 5 ng g^−1^) [34].

In summary, the proposed QuEChERS-based method without a clean-up step was expanded from 218 [41] to 261 analytes, and now proves to be accurate and reliable for the analysis of the ARs and selected PhACs in soil samples, in addition to agricultural pesticides.

### 3.4. Verifying the Method in Different Scenarios

Once the method was validated under the above conditions, it was applied for the analysis of ARs and PhACs in two different scenarios to verify its applicability: Agricultural soils samples irrigated with regenerated water and a soil sample from an environmental forensic investigation of a suspected wildlife poisoning episode.

#### 3.4.1. Agricultural Soil Samples

**Figure 3:**
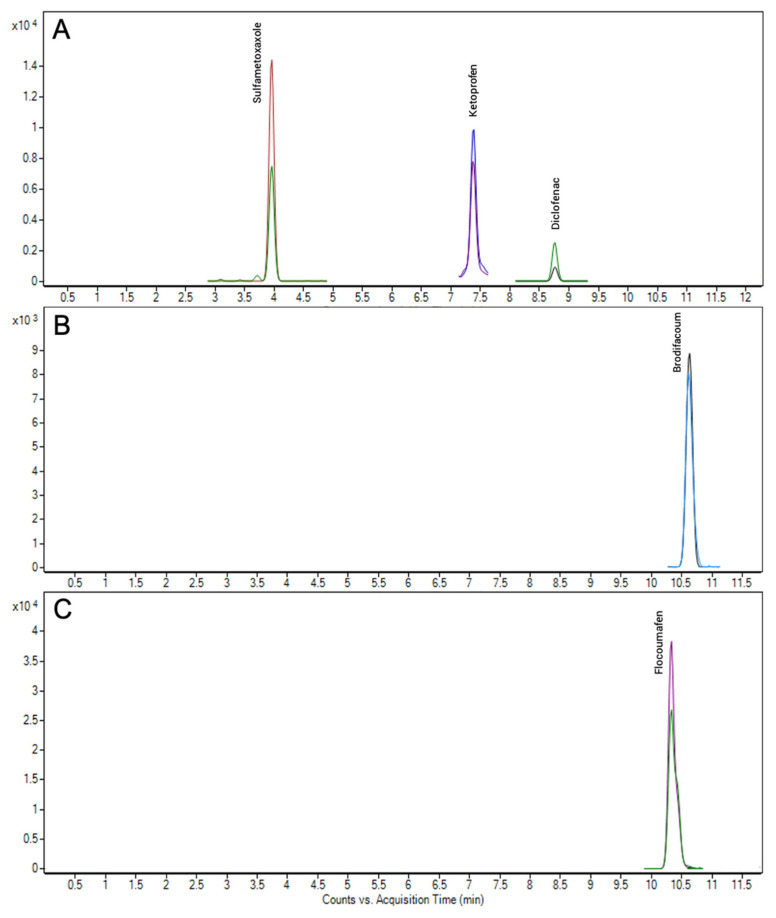
Chromatogram of the regenerated water (**A**) and the two agricultural soils with the identification of (**B**) brodifacoum and (**C**) flocoumafen.

We decided to analyze samples from mid-range farms on the island of Gran Canaria that use regenerated water from the WWTP of the nearby city (Las Palmas de Gran Canaria, 425,000 inhabitants). In the initial analysis of the soil samples, no PhACs residues were detected in any of them, although several pesticide residues from those included in the initial method were detected in the different samples tested (data not shown, see Acosta-Dacal et al., 2021). In addition, thanks to the extension of the analytical scope, brodifacoum was detected in one of the soil samples, and flocoumafen in another (Figure 3, panels B and C), at concentrations of 2.96 and 1.37 ng g^−1^, respectively.

Although these soil samples had been irrigated with regenerated wastewater, no PhACs residues were detected, so there was a possibility that the water was free of residues. In order to verify this, samples of the irrigation water from the WWTP were collected during four consecutive days. These water samples were directly analyzed in the LC-MS/MS where three authorized PhACs were detected: diclofenac, ketoprofen, and sulfamethoxazole (panel A of Figure 3). These PhACs are used for both humans and livestock. In contrast, no AR was found in the water samples. Therefore, the presence of brodifacoum and flocoumafen detected in the soil samples could have been due to a rodent-control application. It should be noted that the water analyzed, although it comes from the same treatment plant, is not the same as the water used in the irrigated plots, since it was subsequently collected. In other words, it is also possible that the water used in the irrigated plots at that time contained the rodenticides and not the PhACs. This possibility is also plausible taking into account that neither brodifacoum nor flocoumafen are EU nonauthorized pesticides as agricultural products (Table 1). 

As mentioned, ARs tend to accumulate in organic matter due to their moderate to high K_OW_, so it is to be expected that most of them are eliminated in the active sludge treatment. This is consistent with what other authors have found, such as Gomez-Canela et al. who determined very few ARs above the detection limit in the effluents of the different treatments of the WWTPs analyzed. Nonetheless, they found flocoumafen in the effluent of the primary (18.1 ng L^−1^) and secondary (18.1 ng L^−1^) treatments from one of the WWTPs and both flocoumafen (29.3 ng L^−1^) and brodifacoum (38.4 ng L^−1^) in the effluent of the tertiary treatment of another one [8]. Therefore, we cannot discard the possibility that although we have not found them in the analyzed water, these compounds could have been present in previous days. On the other hand, the fact that the PhACs detected in water did not show up in the analyzed soil samples could have been due to the low concentrations found, which are very close to the method LOD. The mean concentrations of diclofenac, ketoprofen, and sulfamethoxazole were 1.03, 0.88, and 0.35 ng g^−1^, respectively. These were either below the limit of quantification we reached with our QuEChERS-based extraction method (2.5 ng g^−1^ for both diclofenac and sulfamethoxazole) or slightly higher in the case of ketoprofen (0.5 ng g^−1^). As already mentioned, wastewater content and values may vary on different days even if our samples were in the same range for the period studied.

**Figure 4:**
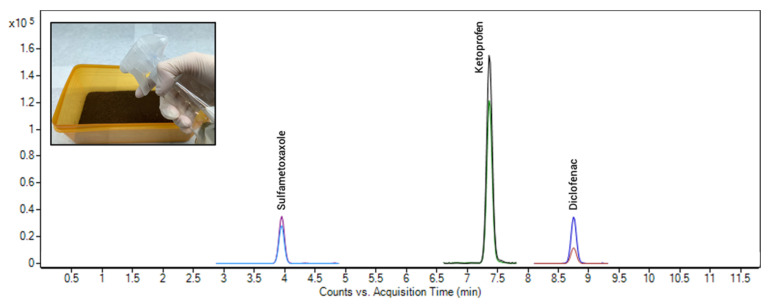
Chromatogram of the soil after the irrigation simulation experiment.

Thus, we decided to investigate whether the soil could accumulate these compounds during irrigation. First, we prepared a pool with the water samples collected during four consecutive days and concentrated it ten times with the intention of achieving a concentration much higher that the LOQ of the three compounds detected. This was performed using a vacuum concentrator RVC 2-25 CD plus (Christ, Germany) at 35 °C and then reconstituting the sample in a volume 10 times lower of ultrapure water. After that, an irrigation simulation model was prepared with the soil used for the validation procedure. Approximately 500 g of soil was placed in a tray container and irrigated with 0.5 L of the concentrated water solution on alternate days for two weeks (Figure 4, inset). Then, the soil was left to air dry for two weeks, homogenized and extracted using the proposed method. Finally, both the concentrated water and the extract were analyzed. Diclofenac ketoprofen and sulfamethoxazole were extracted with an extraction efficiency of 93.4, 85.1, and 107.2%. Therefore, the soil is capable of accumulating these compounds in irrigation if the concentration is higher. A chromatogram of the irrigated soil together with an image of the experiment can be seen in Figure 4.

#### 3.4.2. Environmental Forensic Investigation

**Figure 5:**
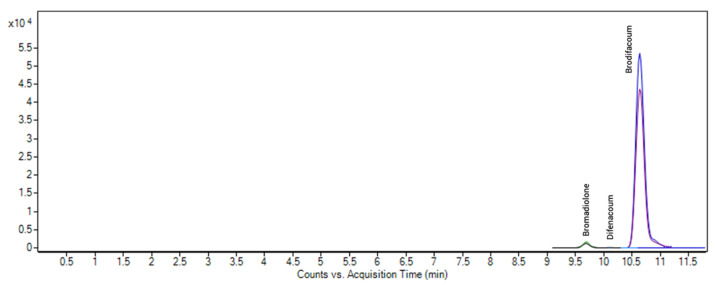
Chromatogram with the identification of brodifacoum, bromadiolone and difenacoum in soil underneath the remains of dead animal in the context of a forensic investigation.

Finally, we tested the validity of the method in another scenario within the context of a judicial forensic investigation. We had dealt with soil samples from a protected natural area on the island of Gran Canaria (Canary Islands, Spain) where the skeletonized remains of several protected birds (ancient corpses) were found. There were strong suspicions that a bait containing some type of poison had been maliciously placed. Therefore, several samples collected in the area were submitted, including a soil sample taken from underneath one of the corpses of a medium-sized bird of prey. This soil sample was analyzed with the method proposed in this article, and as shown in Figure 5, three ARs were identified: brodifacoum, bromadiolone and difenacoum at 223.62, 12.61 and 1.94 ng g^−1^, respectively.

## 4. Conclusions

The proposed methodology was successfully in house validated for the determination of 7 coumarin ARs and 36 PhACs in terms of linearity, trueness, and precision for all the analytes. The LODs were between 0.024 and 6.25 ng g^−1^ and LOQs in the range of 0.5–20.0 ng g^−1^, and only the imipenem presented the typically fixed LOQ for soil residues, 50 ng g^−1^. It was then applied to samples from agricultural plots irrigated with regenerated water, and brodifacoum and flocoumafen were found in two of them. It was also used in a soil sample collected from underneath wildlife carcasses in the context of an environmental forensic investigation where three ARs (brodifacoum, bromadiolone, and difenacoum) were identified.

The scope of this simple method has been extended and now allows the determination of 43 CECs in addition to the 218 pesticides in soil. Thus, it can be used for monitoring agricultural soils and for forensic purposes in soils found under wildlife carcasses. To our knowledge, this is the first time that a QuEChERS methodology is applied to ARs extraction in soil matrix.

## Figures and Tables

**Table 1:** Characterization of target compounds by therapeutical classes or biocides, legal status, and their chromatographic and mass spectrometric conditions.

N°	Compound	Category ^a^	Legal Status in the EU ^b^	tR (min)	Polarity	Quantification		Confirmation		Fragmentor Voltage (V)
						MRM Transition (*m*/*z*)	CE (eV)	MRM transition (*m*/*z*)	CE (eV)	
1	Sulfanilamide	PhACs, AB	Approved	1.06	Positive	173.0 → 93.0	24	173.0 → 76.1	50	126
2	Sulfacetamide	PhACs, AB	Approved	2.28	Positive	215.3 → 92.0	20	215.3 → 65.3	45	90
3	Metronidazole	PhACs, AB	Approved	2.73	Positive	172.1 → 128.0	12	172.1 → 82.1	24	98
4	Sulfadiacine	PhACs, AB	Approved	2.82	Positive	251.0 → 92.0	28	251.0 → 156.0	12	111
5	Sulfapyridine	PhACs, AB	Approved	3.16	Positive	250.0 → 92.0	28	250.0 → 156.0	12	126
6	Sulfameracine	PhACs, AB	Not approved	3.29	Positive	265.0 → 92.0	28	265.0 → 156.0	12	126
7	Sulfametizole	PhACs, AB	Not approved	3.59	Positive	271.0 → 155.9	8	271.0 → 92.0	28	103
8	Sulfametacine	PhACs, AB	Not approved	3.68	Positive	279.1 → 186.0	12	279.1 → 92.0	32	134
9	Sulfametoxipiridacine	PhACs, AB	Not approved	3.76	Positive	281.0 → 155.9	12	281.0 → 92.1	28	121
10	Sulfachloropiridacine	PhACs, AB	Not approved	3.89	Positive	285.0 → 156.0	12	285.0 → 92.1	28	101
11	Sulfametoxazole	PhACs, AB	Approved	3.95	Positive	254.0 → 92.0	28	254.0 → 156.0	12	111
12	Sulfamonomethoxine	PhACs, AB	Not approved	4.02	Positive	281.1 → 92.1	14	281.1 → 156.0	32	120
13	Sulfadoxine	PhACs, AB	Approved	4.13	Positive	311.1 → 156.0	16	311.1 → 92.0	32	126
14	Sulfisoxazole	PhACs, AB	Not approved	4.14	Positive	268.0 → 156.0	8	268.0 → 92.1	24	106
15	Chloramphenicol	PhACs, AB	Approved	4.76	Negative	321.0 → 152.1	4	323.0 → 152.1	4	113
16	Sulfadimetoxine	PhACs, AB	Approved	4.83	Positive	311.0 → 156.0	16	311.0 → 92.0	32	139
17	Sulfaquinoxaline	PhACs, AB	Approved	4.99	Positive	301.0 → 156.0	12	301.0 → 92.1	32	159
18	Cefuroxime axetil (two isomers)	PhACs, AB	Approved	5.40	Positive	533.0 → 447.0	15	533.0 → 386.0	20	160
19	Oxfendazole	PhACs, AH	Approved	5.64	Positive	316.1 → 159.0	32	316.1 → 191.1	16	166
20	Penicillin V	PhACs, AB	Approved	6.48	Positive	383.2 → 159.9	10	383.2 → 113.9	40	130
21	Mebendazole	PhACs, AH	Approved	6.73	Positive	296.1 → 264.1	20	296.1 → 77.0	48	151
22	Cloxacillin	PhACs, AB	Approved	7.09	Positive	468.1 → 159.9	8	468.1 → 177.8	20	126
23	Dexamethasone	PhACs, GC	Approved	7.18	Positive	393.2 → 373.2	2	393.2 → 355.2	6	103
24	Albendazole	PhACs, AH	Approved	7.27	Positive	266.1 → 234.1	16	266.1 → 191.0	32	155
25	Ketoprofen	PhACs, NSAID	Approved	7.36	Positive	255.1 → 209.1	8	255.1 → 77.1	48	123
26	Josamycin	PhACs, AB	Approved	7.52	Positive	860.5 → 173.9	40	860.5 → 108.9	40	200
27	Naproxen	PhACs, NSAID	Approved	7.60	Positive	231.1 → 185.0	10	231.1 → 169.9	13	120
28	Cortiscosterone	PhACs, GC	Approved	7.91	Positive	389.1 → 329.0	13	389.1 → 371.0	13	80
29	Fenbendazole	PhACs, AH	Approved	8.11	Positive	300.1 → 268.1	20	300.1 → 159.0	36	156
30	Flunixin	PhACs, NSAID	Approved	8.11	Positive	297.1 → 279.1	24	297.1 → 264.1	32	141
31	Diclofenac	PhACs, NSAID	Approved	8.75	Positive	296.0 → 215.1	16	296.0 → 214.1	48	103
32	Imipenem	PhACs, AB	Approved	8.75	Positive	300.0 → 125.9	15	300.0 → 98.0	15	50
33	Mefenamic acid	PhACs, NSAID	Approved	9.52	Positive	242.1 → 209.1	28	242.1 → 180.1	44	108
34	Tolfenamic acid	PhACs, NSAID	Approved	9.78	Negative	260.0 → 216.1	8	260.0 → 35.1	20	108
35	Eprinomectin	PhACs, AB	Approved	10.83	Positive	878.5 → 186.0	15	878.5 → 154.0	45	160
36	Moxidectin	PhACs, AH	Approved	11.25	Positive	641.4 → 529.2	5	641.4 → 499.2	5	100
37	Warfarin	ARs	Not approved	7.87	Negative	307.1 → 161.1	20	307.1 → 250.1	20	140
38	Coumatetralyl	ARs	Not approved	8.28	Negative	291.1 → 141.0	30	291.1 → 247.0	20	140
39	Bromadiolone	ARs	Approved	9.74	Negative	525.3 → 250.0	40	527.3 → 250.0	40	200
40	Difenacoum	ARs	Not approved	10.27	Negative	443.2 → 135.0	40	443.2 → 293.0	35	200
41	Flocoumafen	ARs	Not approved	10.36	Negative	541.3 → 382.0	25	541.3 → 161.0	40	230
42	Brodifacoum	ARs	Not approved	10.67	Negative	521.3 → 79.0	50	523.3 → 135.0	45	220
43	Difethialone	ARs	Not approved	10.80	Negative	537.3 → 79.0	50	537.3 → 151.0	45	220
	Cyromazine-d4	P-IS	–	1.58	Positive	171.0 → 86.0	15	171.0 → 129.0	15	100
	Carbendazim-d3	P-IS	–	3.45	Positive	195.1 → 160.1	15	195.1 → 131.9	30	100
	Pirimicarb-d6	P-IS	–	5.12	Positive	245.2 → 78.2	5	245.2 → 185.1	15	70
	Atrazine-d5	P-IS	–	6.66	Positive	221.2 → 179.0	15	221.2 → 101.0	30	90
	Linuron-d3	P-IS	–	7.45	Positive	255.1 → 159.8	15	255.1 → 185.0	15	100
	Coumachlor	P-IS	–	8.52	Negative	341.1 → 161.0	15	341.1 → 284.0	15	120

CE: Collision Energy; tR: Retention time. ^a^ PhACs—pharmaceuticals active compound, ARs—anticoagulant rodenticides, AH—anthelminthic, AB—antibiotic, NSAID—nonsteroidal anti-inflamatory drug, GC—glucocorticoid, P-IS—Procedural Internal Standard ^b^ For human and veterinary drugs, the marketing status in Spain is specified, as shown in the Cima and Cimavet search engine of the Spanish agency for drugs and health products (https://cima.aemps.es/cima/publico/home.html accessed on 8 March 2021; https://cimavet.aemps.es/cimavet/publico/home.html accessed on 8 March 2021). For rodenticides, the legal status reflecting the EU Pesticide Database was considered (https://ec.europa.eu/food/plant/pesticides/eu-pesticidesdatabase/public/?event=activesubstance.selection&language=EN accessed on 8 March 2021), which is valid for the entire EU.—Not applicable.

**Table 2:** Linear studies and threshold limits of the ARs and PhACs.

N°	Compound	Group	Linear Range (ng g^−1^)	R^2^	*p*-Value (Mandel Test) ^a^	LOD (ng g^−1^)	LOQ (ng g^−1^)
1	Sulfanilamide	PhACs	1.56–25	0.9989	0.1611	1.560	2.5
2	Sulfacetamide	PhACs	0.195–50	0.9995	0.5511	0.195	0.5
3	Metronidazole	PhACs	0.195–25	0.9977	0.4488	0.195	0.5
4	Sulfadiacine	PhACs	0.195–50	0.9996	0.4122	0.195	0.5
5	Sulfapyridine	PhACs	0.39–50	0.999	0.2055	0.390	0.5
6	Sulfameracine	PhACs	0.39–25	0.9986	0.9810	0.390	1.0
7	Sulfametizole	PhACs	1.56–25	0.9945	0.2324	1.560	10.0
8	Sulfametacine	PhACs	0.39–25	0.9972	0.1826	0.390	0.5
9	Sulfametoxipiridacine	PhACs	0.39–25	0.9974	0.9264	0.390	0.5
10	Sulfachloropiridacine	PhACs	0.39–25	0.9931	0.4149	0.390	2.5
11	Sulfametoxazole	PhACs	0.195–25	0.9989	0.1782	0.195	2.5
12	Sulfamonomethoxine	PhACs	0.78–50	0.9956	0.0696	0.780	1.0
13	Sulfadoxine	PhACs	0.195–25	0.9996	0.3357	0.195	0.5
14	Sulfisoxazole	PhACs	0.78–25	0.9975	0.8231	0.780	1.0
15	Chloramphenicol	PhACs	6.25–50	0.9900	0.8866	6.250	20.0
16	Sulfadimetoxine	PhACs	0.195–50	0.9998	0.1956	0.195	0.5
17	Sulfaquinoxaline	PhACs	0.78–50	0.9987	0.0582	0.780	1.0
18	Cefuroxime axetil (two isomers)	PhACs	0.39–50	0.9929	0.3722	0.390	1.0
19	Oxfendazole	PhACs	0.39–50	0.9998	0.3931	0.390	0.5
20	Penicillin V	PhACs	1.56–25	0.9987	0.8550	1.560	2.5
21	Mebendazole	PhACs	0.195–50	0.9997	0.1391	0.195	0.5
22	Cloxacillin	PhACs	1.56–50	0.9904	0.1094	1.560	2.5
23	Dexamethasone	PhACs	1.56–50	0.9974	0.0826	1.560	5.0
24	Albendazole	PhACs	0.097–25	0.9969	0.6387	0.097	0.5
25	Ketoprofen	PhACs	0.39–50	0.9985	0.7022	0.390	0.5
26	Josamycin	PhACs	0.39–25	0.9994	0.4666	0.390	1.0
27	Naproxen	PhACs	3.125–50	0.9918	0.3095	3.125	20.0
28	Cortiscosterone	PhACs	1.56–25	0.9974	0.6711	1.560	5.0
29	Fenbendazole	PhACs	0.048–25	0.9979	0.5551	0.048	0.5
30	Flunixin	PhACs	0.097–25	0.9997	0.4675	0.097	0.5
31	Imipenem	PhACs	6.25–50	0.9996	0.2499	6.250	50.0
32	Diclofenac	PhACs	0.097–50	0.9938	0.7354	0.097	2.5
33	Mefenamic acid	PhACs	0.78–25	0.9967	0.1425	0.780	10.0
34	Tolfenamic acid	PhACs	6.25–50	0.9971	0.4907	6.250	10.0
35	Eprinomectin	PhACs	0.78–25	0.9974	0.2500	0.780	2.5
36	Moxidectin	PhACs	1.56–25	0.9972	0.1269	1.560	20.0
37	Warfarin	ARs	0.39–25	0.9941	0.1400	0.390	1.0
38	Coumatetralyl	ARs	0.195–25	0.9977	0.1055	0.195	2.5
39	Bromadiolone	ARs	0.195–25	0.9991	0.1691	0.195	1.0
40	Difenacoum	ARs	0.097–50	0.9943	0.2221	0.097	1.0
41	Flocoumafen	ARs	0.024–50	0.9983	0.2167	0.024	1.0
42	Brodifacoum	ARs	0.097–50	0.9997	0.5200	0.195	1.0
43	Difethialone	ARs	3.125–50	0.9946	0.0797	3.125	20.0

^a^ According to Mandel’s test, the null hypothesis (H0) would indicate that the linear model is adequate to describe the calibration data. Since *p*-value > 0.05 in the range indicated in the table, we accept the H0 and would conclude that the linear model is adequate or reasonable to model the calibration data within this range.

**Table 3:** Recoveries (Rec) and relative standard deviation (RSD).

N°.	Compound	Group	0.5 (ng g^−1^)		1.0 (ng g^−1^)		2.5 (ng g^−1^)		5 (ng g^−1^)		10.0 (ng g^−1^)		20.0 (ng g^−1^)		50.0 (ng g^−1^)	
			Rec (%)	RSD (%)	Rec (%)	RSD (%)	Rec (%)	RSD (%)	Rec (%)	RSD (%)	Rec (%)	RSD (%)	Rec (%)	RSD (%)	Rec (%)	RSD (%)
1	Sulfanilamide	PhACs	N/A	N/A	N/A	N/A	112.1	7.9	90.7	24.2	91.3	17.9	79.9	9.0	63.3	12.8
2	Sulfacetamide	PhACs	83.3	15.1	63.2	10.3	59.8	8.1	78.0	16.5	58.6	5.4	62.8	5.9	75.5	3.7
3	Metronidazole	PhACs	75.3	16.8	86.5	26.9	87.5	7.3	102.5	13.1	94.4	8.0	97.7	5.5	92.7	1.6
4	Sulfadiacine	PhACs	88.1	13.8	76.0	8.2	74.4	5.0	83.8	13.7	75.1	5.1	78.1	6.9	77.6	2.1
5	Sulfapyridine	PhACs	111.0	18.9	78.2	16.1	72.2	5.2	79.2	21.8	75.2	9.7	68.7	7.7	69.7	3.2
6	Sulfameracine	PhACs	N/A	N/A	91.6	19.4	81.3	2.7	85.7	14.7	78.2	5.1	80.5	9.2	78.9	3.7
7	Sulfametizole	PhACs	N/A	N/A	N/A	N/A	N/A	N/A	N/A	N/A	N/A	9.8	71.2	13.4	68.6	3.8
8	Sulfametacine	PhACs	106.8	9.9	84.0	17.8	78.5	11.4	83.9	18.8	78.9	3.2	83.9	7.7	85.4	1.5
9	Sulfametoxipiridacine	PhACs	84.1	10.5	72.0	21.6	62.5	7.8	82.7	17.9	75.5	6.1	74.2	5.8	75.0	2.6
10	Sulfachloropiridacine	PhACs	N/A	N/A	N/A	N/A	81.6	16.2	85.7	22.7	84.3	6.0	87.7	6.7	89.6	2.8
11	Sulfametoxazole	PhACs	N/A	N/A	N/A	N/A	89.3	13.1	96.4	16.7	93.3	7.2	101.2	2.4	103.2	3.0
12	Sulfamonomethoxine	PhACs	N/A	N/A	75.2	10.7	66.6	7.6	80.2	30.7	75.8	4.4	80.3	9.1	80.6	6.1
13	Sulfadoxine	PhACs	96.5	9.7	86.1	6.7	85.2	4.7	96.8	16.0	90.0	6.7	94.0	5.9	89.9	1.4
14	Sulfisoxazole	PhACs	N/A	N/A	89.8	13.9	77.4	15.7	82.8	18.9	71.3	10.6	80.1	7.0	80.8	3.5
15	Chloramphenicol	PhACs	N/A	N/A	N/A	N/A	N/A	N/A	N/A	N/A	N/A	N/A	117.0	11.0	117.8	9.0
16	Sulfadimetoxine	PhACs	85.7	10.3	86.7	7.4	84.8	4.0	91.9	15.6	86.2	6.0	90.9	5.7	88.2	1.3
17	Sulfaquinoxaline	PhACs	N/A	N/A	111.1	8.8	82.9	7.5	84.7	16.6	74.1	5.7	77.0	7.4	78.9	2.1
18	Cefuroxime axetil (two isomers)	PhACs	N/A	N/A	83.4	18.2	96.1	6.8	117.2	11.5	109.5	6.7	115.5	5.3	104.3	0.7
19	Oxfendazole	PhACs	115.1	13.1	83.4	9.8	65.2	2.9	82.3	13.8	69.9	5.3	66.2	4.7	63.4	0.4
20	Penicillin V	PhACs	N/A	N/A	N/A	N/A	66.5	17.6	69.7	26.7	63.0	18.2	65.9	12.8	73.2	9.9
21	Mebendazole	PhACs	92.4	15.4	79.8	13.4	75.1	4.6	96.0	18.0	80.7	5.6	80.7	6.4	79.3	1.5
22	Cloxacillin	PhACs	N/A	N/A	N/A	N/A	76.1	15.4	122.3	18.6	98.7	9.2	101.8	9.7	126.0	11.7
23	Dexamethasone	PhACs	N/A	N/A	N/A	N/A	N/A	N/A	71.1	17.6	68.0	9.1	75.4	6.2	69.9	3.6
24	Albendazole	PhACs	76.3	9.0	76.3	8.2	69.5	3.8	99.5	16.5	82.9	6.3	76.2	5.9	75.8	1.1
25	Ketoprofen	PhACs	103.5	18.3	99.8	13.8	76.6	6.7	93.4	20.7	92.1	6.8	89.6	4.9	87.2	3.2
26	Josamycin	PhACs	N/A	N/A	106.5	14.5	74.5	10.0	80.8	20.2	70.3	7.6	71.8	10.4	74.1	5.6
27	Naproxen	PhACs	N/A	N/A	N/A	N/A	N/A	N/A	N/A	N/A	N/A	N/A	86.9	18.1	94.2	5.2
28	Cortiscosterone	PhACs	N/A	N/A	N/A	N/A	N/A	N/A	112.9	15.4	93.9	6.3	101.3	10.8	99.7	5.4
29	Fenbendazole	PhACs	93.9	12.2	87.1	8.9	83.0	6.2	107.0	14.6	92.2	5.3	90.6	6.2	85.3	1.4
30	Flunixin	PhACs	81.0	11.3	70.2	17.3	68.6	4.2	81.9	17.0	71.8	4.2	69.8	4.2	65.0	2.7
31	Imipenem	PhACs	N/A	N/A	N/A	N/A	N/A	N/A	N/A	N/A	N/A	N/A	N/A	N/A	91.7	17.2
32	Diclofenac	PhACs	N/A	N/A	N/A	N/A	110.3	17.9	107.1	28.8	84.4	17.7	93.9	8.1	85.1	2.1
33	Mefenamic acid	PhACs	N/A	N/A	N/A	N/A	N/A	N/A	N/A	N/A	82.7	14.3	83.1	17.2	82.5	5.9
34	Tolfenamic acid	PhACs	N/A	N/A	N/A	N/A	N/A	N/A	N/A	N/A	121.5	15.4	121.2	7.8	111.5	10.2
35	Eprinomectin	PhACs	N/A	N/A	N/A	N/A	102.1	12.7	104.0	15.4	98.3	11.8	92.1	9.5	84.8	2.6
36	Moxidectin	PhACs	N/A	N/A	N/A	N/A	N/A	N/A	N/A	N/A	N/A	N/A	95.0	14.9	96.3	9.6
37	Warfarin	ARs	N/A	N/A	81.2	9.2	103.8	4.6	106.0	14.7	113.5	6.5	105.5	4.9	85.8	5.0
38	Coumatetralyl	ARs	N/A	N/A	N/A	N/A	104.6	13.5	106.5	12.8	107.3	13.0	99.3	3.3	87.6	5.9
39	Bromadiolone	ARs	N/A	N/A	92.8	14.4	87.5	15.4	90.9	13.8	93.6	7.7	91.4	7.4	82.4	2.6
40	Difenacoum	ARs	N/A	N/A	110.2	12.9	90.6	6.5	110.2	14.3	107.8	5.4	102.6	7.4	86.3	1.4
41	Flocoumafen	ARs	N/A	N/A	105.0	13.0	94.1	16.8	108.1	11.6	108.9	8.0	100.8	5.0	85.1	1.9
42	Brodifacoum	ARs	N/A	N/A	106.5	11.5	87.6	6.5	98.4	14.8	98.0	8.6	93.4	4.2	84.3	4.0
43	Difethialone	ARs	N/A	N/A	N/A	N/A	N/A	N/A	N/A	N/A	N/A	N/A	113.7	4.9	95.7	6.2

## Data Availability

The data presented in this study are available on request from the corresponding author. The data are not publicly available due to some of the data have been obtained from legal proceedings subject to confidentiality.
